# Epigenetic Dynamics in Reprogramming to Dopaminergic Neurons for Parkinson's Disease

**DOI:** 10.1002/advs.202403105

**Published:** 2024-09-16

**Authors:** Byounggook Cho, Junyeop Kim, Sumin Kim, Saemin An, Yerim Hwang, Yunkyung Kim, Daeyeol Kwon, Jongpil Kim

**Affiliations:** ^1^ Laboratory of Stem Cells & Cell Reprogramming Department of Chemistry and Biomedical Engineering Dongguk University Seoul 04620 Republic of Korea

**Keywords:** cellular trajectory, direct reprogramming, induced dopaminergic neuron, Parkinson disease

## Abstract

Direct lineage reprogramming into dopaminergic (DA) neurons holds great promise for the more effective production of DA neurons, offering potential therapeutic benefits for conditions such as Parkinson's disease. However, the reprogramming pathway for fully reprogrammed DA neurons remains largely unclear, resulting in immature and dead‐end states with low efficiency. In this study, using single‐cell RNA sequencing, the trajectory of reprogramming DA neurons at multiple time points, identifying a continuous pathway for their reprogramming is analyzed. It is identified that intermediate cell populations are crucial for resetting host cell fate during early DA neuronal reprogramming. Further, longitudinal dissection uncovered two distinct trajectories: one leading to successful reprogramming and the other to a dead end. Notably, Arid4b, a histone modifier, as a crucial regulator at this branch point, essential for the successful trajectory and acquisition of mature dopaminergic neuronal identity is identified. Consistently, overexpressing Arid4b in the DA neuronal reprogramming process increases the yield of iDA neurons and effectively reverses the disease phenotypes observed in the PD mouse brain. Thus, gaining insights into the cellular trajectory holds significant importance for devising regenerative medicine strategies, particularly in the context of addressing neurodegenerative disorders like Parkinson's disease.

## Graphical abstract

This study explores the reprogramming pathway of directly converted induced dopaminergic neurons for Parkinson's disease treatment, revealing intermediate cell stages and both successful and failed reprogramming trajectories. The histone modifier Arid4b is identified as a key regulator for the successful reprogramming pathway, offering promising insights for regenerative medicine.

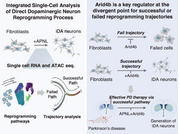

## 1 Introduction

Parkinson's disease (PD) is a debilitating neurodegenerative disorder marked by the gradual loss of dopaminergic neurons in the substantia nigra pars compacta, leading to motor impairments such as tremors, bradykinesia, and postural instability.^[^ ^1^, ^2^, ^3^ ^]^ In recent years, direct lineage reprogramming has emerged as a promising strategy for generating specific neuronal subtypes, including DA neurons, from somatic cells.^[^ ^4^, ^5^ ^]^ This transformative approach holds significant potential by bypassing the reliance on pluripotent intermediates, which not only streamlines the reprogramming process but also mitigates concerns associated with tumorigenicity and immune rejection, thereby offering a safer and more efficient route for cell replacement therapies in neurodegenerative diseases like Parkinson's.^[^ ^6^, ^7^ ^]^ However, the reprogramming pathways into functional DA neurons in particular involves intricate epigenetic changes and transcriptional rewiring, the details of which remain poorly understood.

The trajectory of cell fate conversion during direct reprogramming represents a complex and dynamic process characterized by the sequential activation and repression of lineage‐specific genes.^[^ ^8^ ^]^ This pathway encompasses a series of intermediate stages, each marked by distinct epigenetic signatures and transcriptional programs, ultimately culminating in the acquisition of final cell identity. For example, several lines of study showed that iPSC reprogramming exhibits distinct trajectories, dividing into reprogramming pathway and non‐reprogramming pathways.^[^ ^9^, ^10^ ^]^ Moreover, reprogramming trajectories with district transcriptional networks during the conversion of fibroblasts into induced neurons have been reported.^[^ ^11^, ^12^ ^]^ Also, multiple reprogramming branch points, characterized by unique combinations of transcription factors, are identified along the reprogramming trajectories of induced neurons (iN), induced hepatocytes (iHep), and induced cardiomyocytes (iCM).^[^ ^13^, ^14^, ^15^ ^]^ Furthermore, recent barcode‐based lineage tracing studies have revealed divergent branch points in direct conversion, leading either to successful cell reprogramming or a dead‐end state.^[^ ^16^, ^17^ ^]^ In this study, enhancing the successful reprogramming pathway resulted in highly efficient and effective reprogramming outcomes.^[^ ^18^ ^]^ Taken together, these results suggested that understanding the temporal dynamics and regulatory mechanisms governing this reprogramming pathway is crucial for optimizing reprogramming efficiency and ensuring the fidelity of direct conversion.

In this study, we sought to identify the intermediate epigenetic stages of direct lineage reprogramming into DA neurons, with a focus on identifying key regulatory factors and pathways governing the transition from somatic to DA neuronal identity. By using the single‐cell RNA sequencing (scRNA‐seq) and single‐cell analysis of accessible chromatin (scATAC‐seq), we performed a comprehensive analysis of the reprogramming trajectory at multiple time points, allowing us to delineate the sequential changes in gene expression and chromatin accessibility associated with DA neuronal conversion. Our results reveal a continuous pathway for the reprogramming of DA neurons, characterized by the emergence of distinct intermediate cell populations that play pivotal roles in resetting cellular identity. Through the longitudinal dissection of the reprogramming process, we identified two divergent trajectories: one leading to successful reprogramming and the other culminating in a dead‐end state characterized by incomplete or aberrant DA neuronal differentiation. Interestingly, we identified Arid4b, a histone modifier, as a key regulator at a critical branch point in the reprogramming trajectory, directing the transition toward mature dopaminergic neuronal identity. Overexpression of Arid4b at a branch point significantly enhances reprogramming efficiency, leading to the generation of functional‐induced DA (iDA) neurons capable of ameliorating disease phenotypes in a PD mouse model. Thus, elucidating the epigenetic and transcriptional landscape of DA neuronal reprogramming sheds light on the intricate cellular dynamics underlying direct DA neuronal conversion, providing a foundation for the development of novel regenerative medicine strategies for PD and other neurodegenerative disorders.

## 2 Results

### 2.1 Analysis of Cellular Trajectory in Dopaminergic Neuronal Conversion

**Figure 1:**
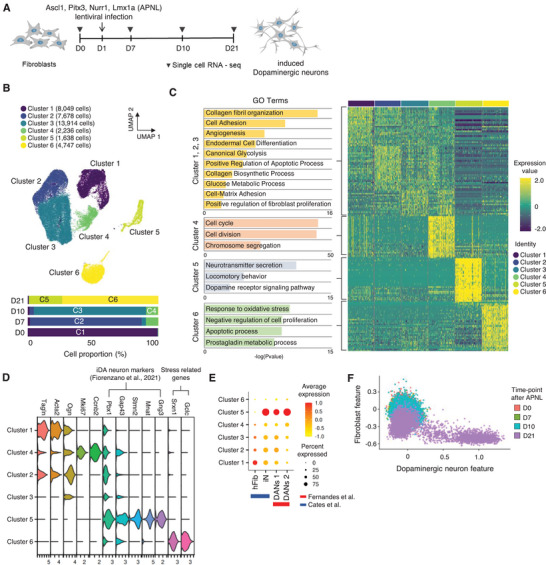
Distinct conversion state of mouse embryonic fibroblasts to dopaminergic neuron by APNL. A) Experimental scheme for studying cellular dynamics of direct neuronal reprogramming by Ascl1, Pitx3, Nurr1, Lmx1a (APNL). B) UMAP visualization of 38262 cells, color‐coded into 6 clusters, from the D0(MEFs), D7, D10, and D21 batch after APNL infection (upper). Cell proportion of clusters across time points (below). C) Hierarchical clustering heatmap of scRNA‐seq data and gene ontology analysis for each cluster. D) A violin plot displaying expression levels of entire clusters through validated markers.^[^ ^39^ ^]^ E) A dot plot validates iDA cluster through module scoring analysis using the public fibroblasts, induced neuron, and dopaminergic neurons markers^[^ ^12^, ^20^ ^]^ F) A Scatter plot showing cell distribution (D0, D7, D10, and D21) across fibroblast and DN features.

The cellular trajectory in DA neuronal reprogramming may be involved in the intricate steps through which cells undergo transformation to adopt specific phenotypes for converting into dopaminergic neurons. Therefore, understanding the cellular trajectory in DA neuronal reprogramming is crucial for refining the reprogramming process. To assess the cellular trajectory during transcription factor‐guided DA neuronal fate conversion, we conducted the single‐cell RNA sequencing at sequential time points on days 0, 7, 10, and 21 with DA neuronal reprogramming based on Ascl1, Pitx3, Nurr1, Lmx1a (APNL) (**Figure** **1**A). Of the 38262 cells analyzed, we found that six clusters were identified to categorize the cell populations during the conversion trajectory: cluster 1 (D0, starting fibroblasts), cluster 2, 3, 4 (D7&10), cluster 5, 6 (D21) as depicted by non‐linear dimensional reduction^[^ ^19^ ^]^ (Figure 1B; Figure S1A, Supporting Information).

To define the molecular features unique to each cluster, we generated an expression heatmap and conducted an analysis of upregulated differentially expressed genes (DEGs) within each cluster, comparing them to the combined remaining five clusters (Figure 1C; Figure S1B, Supporting Information). We observed a significant overlap in Gene Ontology (GO) terms across clusters 1, 2, and 3 during the early stage of reprogramming, including GO terms associated with fibrocytes, such as collagen synthesis and fibril organization, present in all three clusters (Figure 1C). Moreover, Cluster 4, observed 10 days after the reprogramming process, showed enrichment in GO terms related to the cell cycle and chromosome segregation. In the final stage of reprogramming, Cluster 5 exhibited characteristics associated with dopaminergic neurons, while Cluster 6 at the same stage revealed DEGs enriched in apoptotic processes and oxidative stress (Figure 1C,D).

Consistent with these results, we confirmed the expression of representative markers of human midbrain dopaminergic neuron only in cluster 5 (Figure 1D). Further, Cluster 5 exhibited a high correlation with induced neurons^[^ ^12^ ^]^ and iPSCs‐derived dopamine neurons (DANs)^[^ ^20^ ^]^ in the module scoring analysis (Figure 1E). Moreover, we observed DA that was fully reprogrammed ≈21 days after undergoing APNL treatment by conducting time‐point‐dependent Pearson correlation analysis (Figure 1F). Together, these findings reveal a continuous molecular reprogramming pathway leading to induced DA neurons, in which cells transform their identity through distinct intermediate reprogramming stages.

### 2.2 Transition from Cluster 1 to Cluster 3 Drive Erasure of Fibroblast Fate During the Early Stages of DA Neuronal Reprogramming

**Figure 2:**
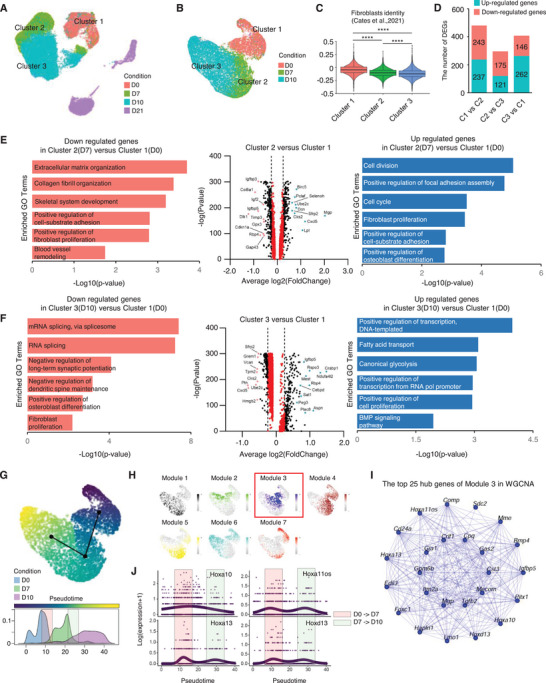
Erasure of fibroblast fate at the early onset of iDA Conversion. A) UMAP plot showing cell distribution across time points of direct reprogramming (D0, D7, D10, and D21). B) UMAP plot showing re‐clustering of sorted cluster 1, 2, and 3 in initial reprogramming (D0, D7, and D10). C) Violin plot displaying gradual reduction of fibroblast module score during initial reprogramming. D) A bar graph showing the total number of differential expression genes based on the comparison of clusters. E) Volcano plot showing differentially expressed genes in Cluster 2 compared with Cluster 1(Wilcoxon rank sum test; two‐sided, log(fc) threshold > 0.25 and log(fc) threshold < −0.25). F) Volcano plot showing differentially expressed genes in Cluster 3 compared with Cluster 1(Wilcoxon rank sum test; two‐sided, log(fc) threshold > 0.25 and log(fc) threshold < −0.25). G) Continuous initial reprogramming trajectory by slingshot‐based UMAP plot (upper). Density plot of initial reprogramming across three‐time points (below). H) Module feature plot showing each co‐expression module colored by each module's uniquely assigned color. I) Network plot showing co‐regulatory top 25 hub genes in the blue module. J) Visualization of smoothed expression patterns of hub genes in blue module, plotted on pseudotime.

Given that progressive erasure of fibroblast identity occurs by the activation of a dopaminergic neuronal program, we initially sought to capture the transcriptomic changes manifesting as cells transition from cluster 1 to 3, including an initial conversion stage. Upon re‐clustering the sub‐sorted clusters 1, 2, and 3, we observed a gradual reduction in fibroblast gene markers as it transitioned into the reprogramming states of clusters indicating loss of fibroblast identity. (**Figure** **2**A,B,C). Importantly, we observed that higher numbers of DEGs were detected in cluster 2 (D7) versus cluster 1 (D0), suggesting that dramatic transcriptional alteration by reprogramming factors occurs during the initial conversion stage (Figure 2D). Consistent with this, gene ontology analysis with down‐regulated genes enriched in Cluster 2 showed non‐neuronal terms, such as collagen fibril organization and fibroblast proliferation (Figure 2E, left panel). In contrast, up‐regulated genes enriched in Cluster 2 correspond to mitotic cell terms including cell division and cell cycle (Figure 2E, right panel) suggesting the mitotic process may be involved in this early reprogramming step. In the comparison between cluster 3 (D10) and cluster 1 (D0), where fibroblast identity erasure has strongly progressed, was enriched metabolic switch terms including fatty acid and canonical glycolysis^[^ ^21^, ^22^, ^23^ ^]^ (Figure 2F, right panel). Pseudotime ordering, conducted through principle curve analysis demonstrated a clear segregation between clusters 1, 2, and 3. This observation provides corroboration for the existence of the distinct states at defined temporal points during initial iDA neuronal reprogramming (Figure 2G).

Subsequently, we employed high‐dimensional Weighted Gene Co‐expression Network Analysis (hdWGCNA) to conduct a more comprehensive exploration of the co‐regulatory gene networks underlying the early stages of induced direct DA neuronal reprogramming. We observed that Modules 3 and 4 were localized to the transition zones between Clusters 1 and 2. Among these, we selected Module 3 for further study in the transition zones because it exhibited mixed cellular identities between Clusters 1 and 2. Additionally, GO term analysis of Module 3 identified significant enrichment in processes such as morphogenesis, development, and transcriptional regulation, consistent with cellular reprogramming (Figure S2A, Supporting Information). In contrast, Module 4 was characterized by fibroblast‐associated gene signatures, including pathways involved in collagen fibril organization and elastic fiber assembly, indicative of a non‐reprogrammed cell population (Figure S2B, Supporting Information). Next, we conducted a screening for hub genes utilizing kME, which relies on epigenetic gene connectivity (Figure 2I). Intriguingly, the Hox gene group (Hoxa10, Hoxa11os, Hoxa13, Hoxd13) was identified as hub genes within module 3, exhibiting increased gene expression levels at the conversion point (Figure 2J). These findings suggest that the Hox gene group is involved in the process of DA neuronal reprogramming due to its regulatory functions in erasing the original cell fate during the process of cell reprogramming.

### 2.3 Divergent Trajectories During the Transition from Cluster 4 to 6 of iDA Conversion

**Figure 3:**
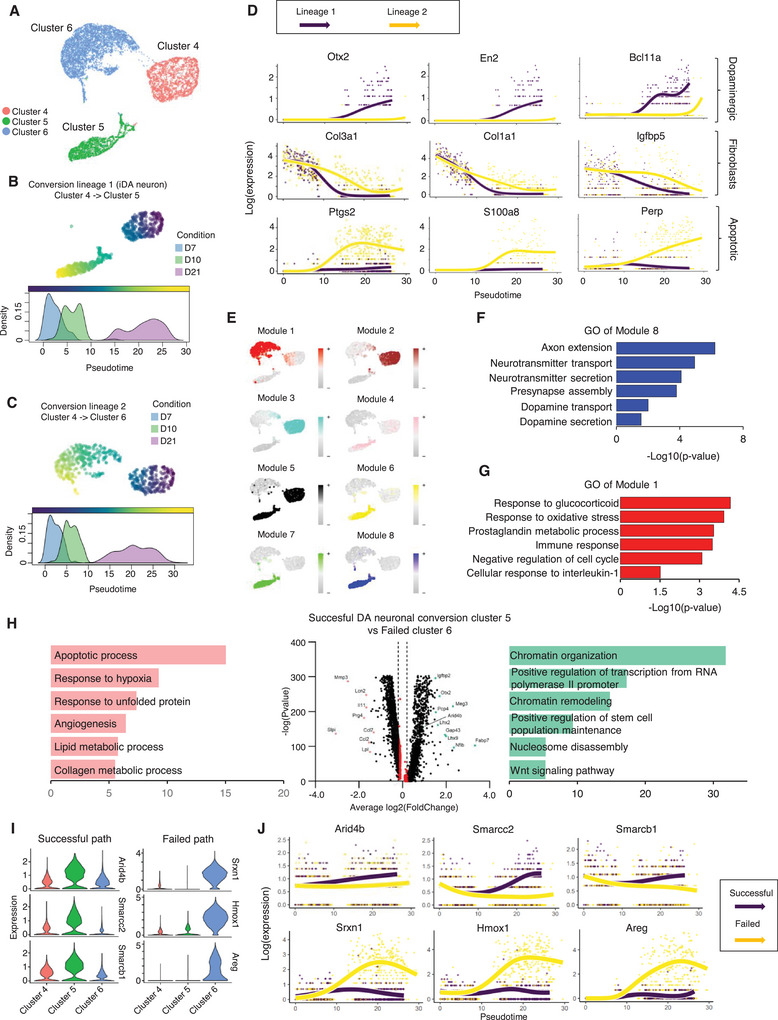
Divergent reprogramming paths during the late stage of iDA conversion. A) UMAP plot showing re‐clustering of sorted clusters 4, 5, and 6 in end point reprogramming (D7, D10, and D21). B) Successful iDA reprogramming trajectory by slingshot‐based UMAP plot (upper). Density plot of Successful iDA reprogramming across three‐time points (below). C) Failed reprogramming trajectory by slingshot‐based UMAP plot (upper). Density plot of failed reprogramming across three‐time points (below). D) Visualization of smoothed expression patterns of representative signatures associated with dopaminergic neuron pathway, fibroblasts, and apoptotic process, plotted on pseudotime. E) Module feature plot showing each co‐expression module colored by each module's uniquely assigned color. F) A bar graph displaying GO terms of the blue module (module 8). G) A bar graph displaying GO terms of the red module (module 1). H) Volcano plot showing differentially expressed genes in Cluster 5 compared with Cluster 6 (Wilcoxon rank sum test; two‐sided, log(fc) threshold > 0.1 and log(fc) threshold < −0.1). I) Violin plot showing the expression level of three chromatin remodelers in the successful path and three representative genes of failed path. J) Visualization of smoothed expression patterns of three chromatin remodelers in successful iDA reprogramming trajectory and three representative genes of failed trajectory.

Next, we examined the reprogramming trajectories in the later stages of DA neuronal lineage conversion. After performing sub‐sorting and pseudotemporal analysis on clusters 4, 5, and 6, we identified two distinct lineage branching points: one from cluster 4 to 5 (Lineage 1) and another from cluster 4 to 6 (Lineage 2), each exhibiting unique transcriptional dynamics (**Figure** **3**A and 3B and C). In particular, lineage 1 exhibited increased expression levels of dopaminergic neuron signatures such as Otx2, En2, and Bcl11a at the reprogramming pathway (Figure 3D). However, lineage 2 revealed highly expressed genes associated with fibroblasts or apoptotic processes (Figure 3D). Additionally, upon conducting hdWGCNA analysis on these clusters, we identified eight modules (Figure 3E). Among them, Module 8, marked in blue, exhibits successful reprogramming originating from lineage 1, illustrating a dopaminergic neuronal network (Figure 3F). Although Modules 6 and 7 were also identified within Cluster 5, Module 6 showed neuron differentiation and development without dopaminergic neuron characteristics, and Module 7 was associated with oxidant detoxification or metabolic processes (Figure S3A,B, Supporting Information). Conversely, Module 1, colored red, represents a failed conversion resulting from lineage 2, predominantly including hub genes associated with immune response and oxidative stress (Figure 3G; Figure S3C,D, Supporting Information). Thus, these data suggest that lineage 1 represents a more successful pathway for reprogramming iDA neurons into cluster 5, while lineage 2 leads to a dead‐end pathway in cluster 6 during the reprogramming process, also indicating the potential role of cluster 4 as a branching point.

Additionally, we conducted cellular transition analysis of induced DA neurons (Cluster 5) and failed reprogramming cluster (Cluster 6) in parallel with normal dopaminergic differentiation processes using scRNA‐seq database of E15.5 and P7 mouse brain (Hook et al., 2018) in the Figure S4A–C, Supporting Information). Notably, we identified that the iDA neuronal signature (Cluster 5) exhibited a high correlation with E15.5 midbrain dopaminergic (mDA) neurons (Figure S4B, Supporting Information). In contrast, the failed reprogramming cluster (Cluster 6) signature did not correlate with either E15.5 or P7 mDA neurons of the mouse brain. (Figure S4C, Supporting Information).

Moreover, to gain a deeper understanding of the molecular features underlying the distinct reprogramming paths, we compared the transcriptional variations between successful and dead‐end trajectories. Specifically, we found that the successful iDA cluster exhibited higher expression levels of epigenetic changes, including chromatin organization, chromatin remodeling, and nucleosome disassembly, compared to the failed cluster (Figure 3H). Similar distinctive differences in transcriptional features between successful iDA clusters and failed clusters were also observed when comparing them to cluster 4 (Figure S5A,B, Supporting Information). For example, specific epigenetic changes, including histone acetylation and methylation, were evident in the upregulated genes associated with successful transitions, such as Lineage 1 (Figure S5A,B, Supporting Information). Consistently, in the successful trajectory, we observed increased expression of epigenetic modifiers such as Arid4b and Smarcc2, while genes related to oxidative stress, like Srxn1, Hmox1, and Areg, exhibited increased expression in the failed trajectory on the later stage of DA reprogramming (Figure 3I,J). Also, we have demonstrated that the expression of all APNL genes is essential to induce both successful reprogramming (Th, Dat, En2, and Arid4b) and failed reprogramming (Srxn1 and Homx1) trajectories for the conversion of iDA neurons (Figure S6A, Supporting Information). Next, we investigated whether Srxn1, Hmox1, and Areg act as failed conversion fate markers or play a key role in leading to failed reprogramming fate. Our results showed that the overexpression of Srxn1, Hmox1, and Areg does not affect the expression of the successful reprogramming fate markers during the direct iDA neuronal reprogramming process (Figure S7A, Supporting Information). This result indicates that Srxn1, Hmox1, and Areg are solely markers of failed reprogramming and do not influence the successful reprogramming trajectory. We further found that representative markers expression of Cluster 6 showed mixed cellular identities including kerationocytes and melanocytes (Figure S8A,B, Supporting Information). This cluster does not exhibit the typical markers of mature dopaminergic (DA) neurons, which explains the absence of obvious iDA neuronal markers in this cluster. Thus, this finding indicates that the failure trajectory, cluster 6, represents a cell population that diverges toward a dead‐end pathway, characterized by signals of cell death or an indeterminate type lacking a specific identity. Consequently, it is essential to identify conditions that effectively promote accurate and successful reprogramming.

### 2.4 Alterations in Chromatin Accessibility During iDA Neuronal Reprogramming

**Figure 4:**
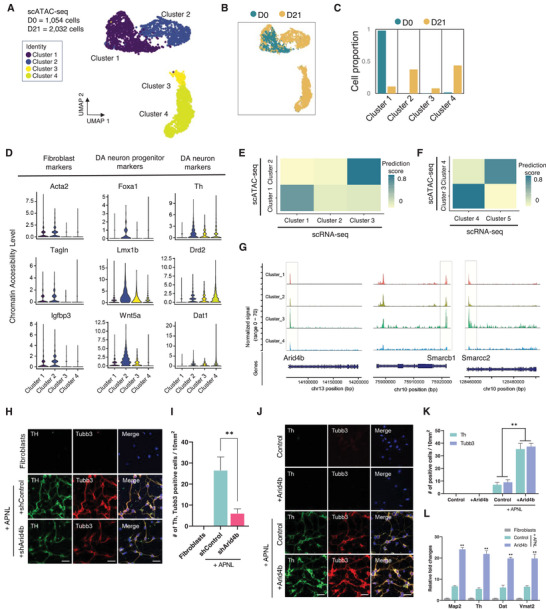
Single‐cell chromatin accessibility landscape in iDA conversion. A) UMAP visualization of 3086 cells in scATAC‐seq, color‐coded into five clusters, from the D0(MEFs), D7 batch after APNL infection. B) UMAP plot of scATAC‐seq displaying cell distribution across time points of direct reprogramming (D0, D7). C) Cell proportion of clusters across time points (D0, D7). D) Violin plot showing chromatin accessibility level of validated genes associated with pan‐fibroblast, dopaminergic neuron progenitor, and mature dopaminergic neuron markers. E) Heatmap showing annotation of scATAC‐seq clusters (Cluster 1, 2) based on label transfer of scRNA‐seq dataset (Cluster 1, 2, and 3). F) Heatmap showing annotation of scATAC‐seq clusters (Cluster 3, 4) based on label transfer of scRNA‐seq dataset (Cluster 4, 5). (G) Coverage plot showing normalized open chromatin signal in each cluster on Arid4b, Smarcb1, and Smarcc2 gene track. H) Immunofluorescence of DA neuronal marker Th and neuronal maker Tubb3 in iDAs at 20days after Ascl1, Nurr1, Pitx3, and Lmx1a (collectively, ANPL) along with shArid4b infection. Scale bar = 50 µm (Left). I) The bar chart shows the quantification data of Figure 4H. J) Immunofluorescence of DA neuronal marker Th and neuronal maker Tubb3 in iDAs at 20 days after FUW‐APNL along with FUW‐Arid4b infection. Scale bar = 50 µm. K) The bar chart shows the quantification data of Figure 4K. L) qRT‐PCR analysis of DA neuronal markers (Map2, Th, Dat, and Vmat2). Data represent mean ± SEM; two‐tailed Student's *t*‐test, ^*^ *p* < 0.05 and ^**^ *p* < 0.005 (*n* = 3, independent samples per group).

To understand the alterations in chromatin accessibility necessary for successful reprogramming of DA neurons, we conducted scATAC sequencing on fibroblasts and reprogrammed DA neurons at 21 days. First, we obtained 1054 and 2032 cells respectively, and merged objects by quantifying the unified peak set in each dataset. We identified four distinct clusters and confirmed that the conversion trajectory was clearly divided throughout the reprogramming process (**Figure** **4**A–C; Figure S9A, Supporting Information). Based on chromatin accessibility levels of each marker gene, Cluster 2 showed characteristics of fibroblasts and dopaminergic progenitors, while Clusters 3 and 4 had higher open chromatin levels associated with mature DN features compared to Cluster 2 (Figure 4D; Figure S9B, Supporting Information). In contrast, Cluster 1 only exhibited fibroblast characteristics, primarily detected at Day 0 (Figure 4D). To gain a deeper understanding of the distinctive epigenetic characteristics of these clusters during direct neuronal reprogramming, we integrated the transcriptional and epigenetic dimensions of each cluster using the previous method.^[^ ^24^ ^]^ After comparing clusters, we found distinct correlations between scRNA‐seq and scATAC‐seq in these clusters (Figure 4E,F; Figure S9C,D, Supporting Information). These results indicate the presence of distinct clusters with unique transcriptional and epigenetic traits during the reprogramming of dopaminergic neurons.

Additionally, we observed dynamic changes not only in the epigenetic levels of Arid4b during iDA reprogramming but also an increase in the accessibility of Arid4b within Cluster 3 of scATAC‐seq identified as a branch point, indicating its significant role in determining the divergent trajectory of direct DA neuronal reprogramming (Figure 4G). In order to verify the significance of Arid4b in successful iDA generation in the divergent trajectory for direct conversion, we conducted Arid4b shRNA knockdown experiments in vitro with lentiviral APNL for iDA reprogramming. Indeed, suppression of Arid4b at the later stage of iDA reprogramming led to a significant reduction in the number of Th and Tubb3 positive cells, however suppressing Arid4b did not influence the basal generation of iDNs in response to APLN factors expression alone (Figure 4H,I). Next, we investigated the consequences of increased Arid4b expression at the later stage of direct DA neuronal reprogramming. We confirmed that overexpressing Arid4b led to a significant increase in the number of Th‐positive and Tubb3‐positive cells (Figure 4J,K). Consistently, we observed a significant transcriptional upregulation of the DA neuronal markers such as Map2, Th, Dat, and Vmat2 (Figure 4L). These findings strongly indicate that Arid4b plays a crucial role in facilitating epigenetic changes during the divergent trajectory and is associated with successful reprogramming.

### 2.5 Arid4b Plays a Crucial Role in the Divergent Trajectory for Successful In Vivo DA Neuronal Reprogramming

**Figure 5:**
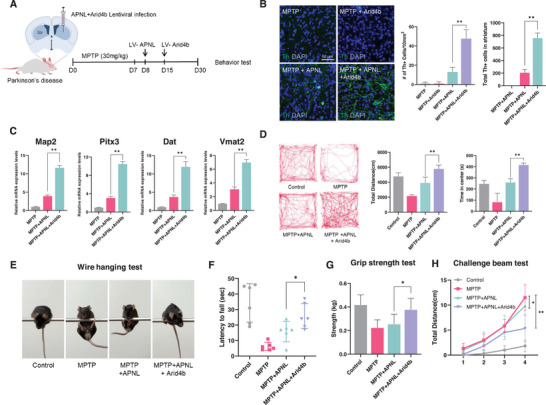
Arid4b overexpression efficiently rescues Parkinson's disease phenotypes in MPTP‐induced PD mice by in vivo direct reprogramming. A) The schematic diagram depicts the procedures for direct reprogramming into induced dopaminergic neurons using Arid4b overexpression in the MPTP‐PD model. B) Representative image of the Th staining in the stratum treated with MPTP, MPTP/APNL, MPTP/Arid4b, and MPTP + ANPL/Arid4b. Scale bar = 25 µm (Left). The bar chart shows the quantification data (Right). C) qRT‐PCR analysis of DA neuronal markers (Map2, Pitx3, Dat, and Vmat2) in the APNL with Arid4b overexpressed in the striatum of the MPTP PD mouse. Expression levels are normalized to Gapdh. D) Digitally tracking the distance traveled and time spent in the central zone by mice subjected to the open field test. E) MPTP PD mouse behaviors after overexpression of APNL with Arid4b in the wire‐hanging test. Different views of a mouse during the test. F) The latency to fall in the wire‐hanging test. G) Values were measured using the forelimb grip strength test in mice treated with MPTP, MPTP/APNL, MPTP/Arid4b, and MPTP + ANPL/Arid4b. H) Number of errors per step on the challenging beam traversal in Control (Saline), MPTP, MPTP + ANPL, and MPTP + ANPL + Arid4b. Data represent mean ± SEM; two‐tailed Student's *t*‐test, ^*^ *p* < 0.05 and ^**^ *p* < 0.005 (*n* = 3, independent samples per group).

Next, we explored whether Arid4b is pivotal in the divergent trajectory leading to successful in vivo DA neuronal reprogramming. To assess this, we established a mouse model of Parkinson's disease (PD) induced by 1‐methyl‐4‐phenyl‐1,2,3,6‐tetrahydropyridine (MPTP). Following 12 days of APLN delivery, lentiviral particles containing Arid4b were additionally injected into the striatum of MPTP‐treated mice (**Figure** **5**A). To directly investigate the effects of Arid4b on the lineage reprogramming of iDA neurons in vivo, we examined Th‐positive cells in the mouse striatum. Mice overexpressing Arid4b in the later stage of the APNL‐mediated reprogramming process showed a significant increase in the number of Th+ cells compared to MPTP‐treated controls or mice infected with APNL alone (Figure 5B). Similarly, quantitative reverse transcription (qRT)‐PCR analysis demonstrated increased transcriptional expression of Map2, Pitx3, Dat, and Vmat2 in Arid4b overexpressing APNL mice compared to control groups (MPTP alone or MPTP and APLN factors) (Figure 5C).

Moreover, we conducted the open field test to evaluate spontaneous locomotion and examine the motor function of these mice. Strikingly, mice overexpressing Arid4b in the APNL‐mediated in vivo reprogramming exhibited a remarkable restoration of movement compared to the control groups. This was evidenced by an increase in total distance moved and a higher proportion of overall activity in the center zone of the open field (Figure 5D). In the wire‐hanging test, Arid4b overexpression in the APNL‐treated mice showed also an increased wire‐hanging time compared to the control mice (Figure 5E,F). Similar results were observed in the forelimb grip strength test, where Arid4b overexpression with APNL‐treated mice exhibited increased forelimb grip strength compared to the control mice (Figure 5G). Furthermore, Arid4b overexpression in the APNL treated mice displayed a significant decrease in stepping errors in the challenge beam test compared to the control mice (Figure 5H)

Additionally, we evaluated the effectiveness of Arid4b‐mediated in vivo DA reprogramming for reversing the phenotypes in a second PD model, where one hemisphere of the midbrain is damaged with 6‐hydroxydopamine (6‐OHDA). After 6‐OHDA treatment, lentiviral particles of Arid4b were additionally injected into the APLN‐treated striatum, as in the MPTP model (Figure S10A, Supporting Information). Notably, Arid4b in the later stage of APNL‐mediated in vivo reprogramming in the mice resulted in a larger number of Dat+ and Th+ iDA neurons in the striatum (Figure S10B,C, Supporting Information). Also, we confirmed that iDA total neurons were efficiently induced by APNL with Arid4b compared to only APNL expression (Figure S10D, Supporting Information). To assess the motor functions and muscle strength of these mice, we conducted the wire‐hanging test to evaluate the strength of the forelimbs. We observed increased wire hanging time in mice overexpressing Arid4b with APNL compared to overexpressing only APNL (Figure S10E, Supporting Information). Additionally, Arid4b overexpression in APNL‐treated 6‐OHDA mice resulted in increased forelimb grip strength compared to control mice (Figure S10F, Supporting Information). We also conducted a cylinder test to evaluate forelimb asymmetry, where Arid4b overexpression in APNL‐treated 6‐OHDA mice exhibited increased rearing rates (Figure S10G, Supporting Information). Overall, these findings suggest that Arid4b plays a crucial role in the divergent pathways leading to the successful in vivo reprogramming of DA neurons, significantly improving lineage reprogramming efficiency, and rescuing Parkinson's disease phenotypes in mouse models of PD.

## 3 Discussion

Identifying the reprogramming trajectory is crucial for advancing our understanding of cellular reprogramming, optimizing reprogramming protocols, and ensuring the quality and safety of reprogrammed cells for therapeutic purposes.^[^ ^25^, ^26^, ^27^ ^]^

In this study, we utilized scRNA‐seq and scATAC‐seq to dissect the intricate cellular dynamics and epigenetic changes involved in the direct lineage reprogramming of somatic cells into induced dopaminergic (iDA) neurons. This approach allowed us to capture the reprogramming trajectory at multiple time points, providing a detailed map of the cellular states and transitions that occur during the reprogramming process. Our analysis revealed a continuous reprogramming pathway characterized by the emergence of distinct intermediate cell populations. These intermediate stages are crucial for resetting the host cell fate and facilitating the transition toward a dopaminergic neuronal identity. Specifically, we identified six clusters of cells at different stages of reprogramming: starting fibroblasts (Day 0), early reprogramming stages (Days 7 and 10), intermediate and final stages of successful reprogramming (Day 21), and cells in a dead‐end state characterized by apoptotic processes and oxidative stress (Day 21). Through longitudinal dissection, we identified two distinct reprogramming trajectories. The successful reprogramming pathway leads to the generation of functional iDA neurons, with cells in this trajectory exhibiting increased expression of dopaminergic neuron markers such as Otx2, En2, and Bcl11a. This pathway is associated with specific epigenetic changes, including chromatin remodeling and histone modifications. Conversely, the dead‐end pathway results in incomplete or aberrant DA neuronal differentiation, with cells showing high expression of genes associated with fibroblasts and apoptotic processes, characterized by increased oxidative stress and immune response‐related gene expression. These findings provide valuable insights into the cellular and molecular mechanisms underlying direct lineage reprogramming into DA neurons.

A key finding of our study is the identification of Arid4b, a histone modifier, as a crucial regulator at a critical branch point in the reprogramming trajectory. While previous studies have explored the various transcription factors and epigenetic modifiers in DA reprogramming, we identified additional regulatory factor to improve DA reprogramming outcomes based on the precise molecular mechanisms underlying the observed reprogramming trajectories. Previously, Arid4b was found to physically and functionally interact with the HDAC1 and Sin3a complex.^[^ ^28^ ^]^ Consistently, deficiency in Arid4b resulted in an increase in H3K27me3 and a decrease in H3K27Ac levels at key developmental gene locations during pluripotent reprogramming. These studies indicate the distinct role of Arid4b in cell fate decisions during the reprogramming processes. In this regard, manipulation of Arid4b expression levels significantly enhanced in vivo direct reprogramming efficiency and effectively reverses disease phenotypes observed in PD mouse models, indicating Arid4b possibly orchestrates the transition toward successful dopaminergic neuronal identity through histone methylation in the later state of DA reprogramming. Thus, this result not only highlights the therapeutic potential of targeting specific regulators but also underscores the importance of understanding the molecular mechanisms underlying reprogramming trajectories. However, despite these results, our study showed that the factors driving these divergent outcomes remain elusive, underscoring the complexity of the reprogramming process. Moreover, future work will be essential to understanding how Arid4b lead to a successful trajectory in DA reprogramming.

Overall, our results underscore the importance of elucidating the temporal dynamics and regulatory mechanisms governing the reprogramming trajectory. By gaining insights into the intricate cellular dynamics underlying direct DA neuronal conversion, we provide a foundation for the development of novel regenerative medicine strategies for PD and other neurodegenerative disorders. The enhanced understanding of epigenetic and transcriptional landscapes presented here holds significant promise for the optimization of reprogramming efficiency and the development of more effective therapeutic interventions aimed at addressing the challenges posed by PD.

## 4 Experimental Section

### Ethics Statement

All experimental procedures and animal care in this study were conducted in compliance with the guidelines of the Institutional Animal Care and Use Committee at Dongguk University.

### Lentivirus Generation

Following established protocols,^[^ ^29^, ^30^, ^31^, ^32^, ^33^ ^]^ lentiviruses were generated by cultivating HEK293T cells in DMEM supplemented with 10% FBS and 1% P/S. A day before transfection, 2 ×10^7^ cells were seeded in a 15 cm culture dish. The next day, cells were transfected with lentivirus constructs (FUW‐Ascl1, FUW‐Nurr1, FUW‐Pitx3, FUW‐Lmx1a, FUW‐Arid4b) using calcium phosphate coprecipitation. The medium was replaced 24 h post‐transfection, and viruses were collected 72 h later. Supernatants containing the lentivirus were centrifuged to remove cell debris, filtered through a 0.45 µm filter, pelleted by ultracentrifugation, and resuspended in cold PBS. Lentivirus titers were determined using FUW‐GFP to monitor GFP expression and calculate the multiplicity of infection. The virus can be titrated based on the GFP reporter (FUW‐GFP) as previously reported.^[^ ^30^, ^34^ ^]^ HEK cells were seeded in a 24‐well plate at a density of 7 × 10^3^ to 10^4^ cells per well. Cells reached ≈70% confluency the next day at the time of titration. A serial dilution (1:10, 1:100, 1:1000) of the viral supernatant was prepared using HEK culture medium. After 8 h of exposure to the lentivirus, the medium was changed. The proportion of GFP‐positive cells was assessed 48–72 h post‐transduction. In undiluted wells, over 90% of the cells were expected to be GFP‐positive, while in wells with a 10‐fold dilution, this percentage was anticipated to exceed 30%. This method yielded lentivirus titers of 1 × 10^8^ transducing units per milliliter (TU/mL).

### scRNA‐Seq Library Construction and Sequencing

The 10X Genomics scRNA‐seq protocol was followed for the isolation of pure cells for single‐cell RNA sequencing (10X Genomics, CG00054). Cell preparations were performed according to the previous protocol.^[^ ^35^, ^36^ ^]^ In brief, cells at various time points throughout the iDA reprogramming process were harvested and resuspended at a concentration of 1 × 10^6^cells mL^−1^ in 1X PBS with 0.5% BSA. The cell suspension was combined with an equal volume of 0.5% BSA in 1X PBS and filtered through a 40 µm strainer. The filtered cells were then counted using a hemocytometer and diluted to a concentration of 1000 cells µL^−1^. These cells were subsequently captured as single cells using the 10X Genomics Single‐Cell 3′ platform. The 10X library preparation protocol was followed exactly as recommended. Single‐cell cDNA libraries were pooled and sequenced with 5 million reads per sample using a HiSeq 4000 sequencer.

### scATAC‐Seq Library Construction and Sequencing

Using the Chromium Single Cell ATAC User Guide as a reference, scATAC‐seq libraries were prepared. The process began with isolating cell nuclei via the Chromium Nuclei Isolation Kit for Single Cell ATAC sequencing (10X Genomics, CG000505). Nuclei were then loaded into a Chromium Instrument (10X Genomics) at 3000 nuclei µL^−1^ to create single‐cell gel beads in emulsion (GEMs), with a goal of recovering 2000 nuclei. Afterward, scATAC‐seq libraries were generated using the appropriate Chromium kits, including the Single Cell ATAC Kit, Gel Bead Kit v2, and i7 Multiplex Kit (10X Genomics). Sequencing was conducted on the Illumina Novaseq SP platform. To minimize batch effects, all scATAC‐seq libraries were prepared at the same time and sequenced on the same lane.

### Bioinformatic Analysis—Data Alignment and Quality Control for scRNA‐Seq

A 10X Genomics CellRanger v6.1.2 software to analyze all datasets was used. Fastq files were mapped and aligned to the mouse genome (mm10) using STAR software with default settings. Cells were filtered out if they had more than 10% mitochondrial gene content, fewer than 200 unique feature counts, or an nCount RNA below 40000.

### Bioinformatic Analysis—Alignment and Quality Control of scATAC‐Seq Data

The Cellranger ATAC pipeline (version 1.2.0) was employed to preprocess the data resulting from sequencing. Initially, Tn5 sites were aligned to the mm10 (mouse genome), duplicate reads were removed, and background cells were filtered out using the “cellranger‐atac count” function. This process produced barcoded fragment files, which were then imported into Signac (version 1.3.0),^[^ ^24^ ^]^ an R package compatible with R version 4.0.2 (R core team, 2021), for downstream analysis using the standard Signac/Seurat pipeline. The nucleosome signal strength and TSS (Transcription Start Site) enrichment for each cell were calculated using the “NucleosomeSignal” and “TSSEnrichment” functions in Signac, respectively. Cells that were outliers in the quality control (QC) metrics were excluded following the standard processing protocols of Signac.

### Bioinformatic Analysis—Dimensionality Reduction and Clustering of scRNA‐Seq

To combine the datasets (D0, D7, D10, and D21) and detect transcriptional changes during iDA neuronal reprogramming, an integrated analysis was performed using the IntegrateData function of the Seurat package (v4.3.0).^[^ ^19^, ^36^ ^]^ The non‐linear dimensional reduction algorithm “RunUMAP” and clustered the cells based on variable genes was applied. The AddModuleScore function from the Seurat package was used to score the iDA cluster by subtracting the expression levels of published iN and iDA neuron signatures.^[^ ^12^, ^20^ ^]^

### Bioinformatic Analysis—Dimensionality Reduction and Clustering of scATAC‐Seq

To perform dimensionality reduction, Latent Semantic Indexing (LSI) was applied using Signac's “RunTFIDF” and “RunSVD” functions. Dimensions with a high correlation to read depth were identified with the “DepthCor” function in Signac and were then excluded from subsequent analysis. To determine the number of LSI dimensions to utilize, Signac's “ElbowPlot” function and manual inspection were employed. UMAP hyperparameters were adjusted to ensure consistent object shapes, a process executed using R version 4.0.2. Once the appropriate hyperparameters were determined, Signac/Seurat's “RunUMAP” function was applied to the previously selected LSI dimensions to generate the UMAP embedding. The “FindNeighbors” function in Signac/Seurat, which uses the same LSI dimensions as UMAP, was employed to compute the nearest neighbors graph. Following this, the “FindClusters” function in Signac/Seurat was run at different resolutions. Clusters were labeled as Cluster1, Cluster2, Cluster3, and Cluster4 by assessing accessibility near known marker genes for fibroblasts and dopaminergic neurons using the “ViolinPlot” function in Signac. Also, Normalized chromatin accessibility signal of Arid4b, Smarcb1, and Smarcc2 genes were projected to chromosome track using Signac's “CoveragePlot” function, which analyzed peaks within 100 kb of the gene of interest.

### Bioinformatic Analysis—Molecular Dynamics and Pseudotime Analysis During iDA Reprogramming

Slingshot was employed for conducting trajectory analysis. Initially, the dimensional reduction was performed using the Multidimensional Scaling (MDS) of subset data, encompassing time‐points D0‐D10 and D7‐D21. Next, Principal Component Analysis (PCA) was performed on both individual and integrated datasets. The Seurat objects were converted into SingleCellExperiment objects. Slingshot trajectory analysis was then conducted using the clustering information from Seurat along with the dimensionality reduction results obtained from MDS.

### Bioinformatic Analysis—Analyzing Differential Gene Expression

Differentially expressed gene signatures were identified using the likelihood‐ratio test with the FindMarkers function (ident.1 and ident.2) and visualized through volcano plots. To find DEGs in each time‐point cluster, expression levels were compared using the FindMarkers function with parameters set to min.pct = 0.1, logfc.threshold = 0.2, and *p*‐value < 0.05.

### Bioinformatic Analysis—Gene Co‐Expression Network Analysis Using hdWGCNA

The hdWGCNA package^[^ ^37^ ^]^ was applied to analyze gene co‐expression networks in clusters during iDA neuronal reprogramming. After configuring the Seurat object for WGCNA, metacells were generated with the following parameters: assay = “SCT”, max_shared = 20, min_cells = 300, and reduction = “umap”. Metacells were created by pooling only those cells that belonged to the same cell type within the same sample. The ConstructNetwork function was then used to build the co‐expression network of the top 25 hub genes.

### Bioinformatic Analysis—Integrative Analysis of scRNA‐Seq and scATAC‐Seq Data

The integration of scRNA‐seq and scATAC‐seq was performed using the Seurat and Signac packages, as described by.^[^ ^24^ ^]^ To begin, “anchors” were established between the scRNA‐seq and scATAC‐seq experiments by estimating the transcriptional activity of genes. This was done by quantifying ATAC‐seq counts within the 2 kb upstream region and the gene body using the GeneActivity() function from the Signac package. The gene activity scores obtained from the scATAC‐seq data were then used for canonical correlation analysis (CCA) alongside gene expression data from the scRNA‐seq. By identifying these anchors, annotations from the scRNA‐seq dataset were transferred to the scATAC‐seq cells. This process resulted in the scATAC‐seq cells receiving predicted annotations from the scRNA‐seq dataset, which were stored in the predicted.id field.

### Animals

All animal experiments received approval from the Institutional Animal Care and Use Committee at Dongguk University (IACUC‐2021‐042‐2) and were carried out according to institutional guidelines. Twelve‐week‐old male C57BL/6 mice, weighing between 25 and 29 g, were obtained from Central Laboratories Animal Inc., Korea, and used in the study. The mice were kept on a 12 h light/dark cycle (lights on from 9:00 to 21:00) with free access to food and water. The Animal Care Committee at Dongguk University also approved all experimental procedures (IACUC‐2019‐043‐2), and these procedures followed the university's standards for animal care and use. Mice were randomly assigned to experimental groups, and all data were analyzed under blinded conditions.

### In Vivo Direct Lineage Reprogramming in Parkinson's Disease Mouse Model

All animal procedures were approved by the Committee on Animal Welfare at Dongguk University and conducted following the institutional ethical guidelines. These procedures complied with the standards set by the Association for Assessment and Accreditation of Laboratory Animal Care. Following established protocols^[^ ^35^ ^]^ in the mouse studies, 12‐week‐old male C57Bl/6 mice were randomly assigned to receive either 6‐OHDA/MPTP or saline injections. For the 6‐OHDA model of Parkinson's disease, the mice were anesthetized with avertin and secured in a stereotaxic frame. A 6‐OHDA solution (10 µg µL^−1^ in 0.02% ascorbate/saline) was injected into the substantia nigra at specific coordinates (AP −3.1 mm, ML ±1.1 mm, DV −4.4 mm) at a rate of 0.5 µL min^−1^ over 4 min using a 26‐gauge 10 µL^−1^ Hamilton syringe. Three days later, a lentivirus encoding APLN with Arid4b was injected into the striatum at coordinates AP +0.4 mm, ML ±1.5 mm, and DV −2.8 mm. Based on the in vitro results, which yielded lentivirus titers of 1 × 10^8^ TU mL^−1^, the viral supernatant was diluted in PBS to achieve the desired concentration. A viral solution with a concentration of 1 × 10^8^ TU mL^−1^ was prepared in a total volume of 200 µL. From this solution, 4 µL (equivalent to 4 × 10^5^ TU) was injected into the striatum. For the MPTP Parkinson's disease model, mice were administered MPTP at a dose of 30 mg k^−1^g via intraperitoneal injection over the course of 7 days. A week following the MPTP injection, a suspension of the APNL with Arid4b lentivirus was stereotaxically injected into the bilateral striatum of the mice. The injection was carried out at coordinates anteroposterior (AP) +0.4 mm, mediolateral (ML) ±1.5 mm, and dorsoventral (DV) −2.8 mm.

### Behavior Test—Open Field Test

The overall locomotor activity levels of the mice were measured using the open field test, which consisted of a square arena (40 × 40 × 40 cm) equipped with a tracking system.^[^ ^38^ ^]^ Each mouse was gently placed in the center of the arena, and recording commenced immediately. The mice were allowed to move freely for 10 min, and their movement and exploration behaviors were analyzed using the Smart (Ver.3.0) software.

### Behavior Test—Wire‐Hanging Test

The wire‐hanging test was used to evaluate neuromuscular abnormalities of motor strength. Mice were placed on an elevated wire, and the time it took for them to fall was recorded as the latency time.

### Behavior Test—Grip Strength Test

A Grip Strength Meter (JD‐A‐22, Jeung‐do, Korea) was utilized to measure the forelimb grip strength of a mouse. The maximum pull force, recorded in grams, was monitored on a digital force transducer as the mouse grasped the bar.

### Behavior Test—Challenging Beam Test

The beam traversal test, designed to evaluate motor coordination and balance, was administered to mice. This test utilized a 1 m^2^ plastic beam divided into four sections, each 25 cm in length. The sections tapered from a diameter of 4 to 1 cm. Mice were initially trained to cross the beam from the narrow end into their home cage over two days, with three trials conducted each day. On the third day, a mesh grid, fitted to each beam width, was installed above the beam. The grid either aided or hindered the mice's footing. The traversal of each mouse was video recorded until it placed a forelimb into the home cage. The footage was then analyzed in slow motion to count the number of foot slips on the beam.

### Behavior Test—Cylinder Test

To assess forelimb asymmetry, the cylinder test was utilized. Mice were placed in a cylinder that was 30 cm tall and 20 cm in diameter for 3 min, and the number of times their paws touched the wall was recorded. The touches were categorized into simultaneous touches, independent touches with the left or right paw, and free touches.

### Statistical Analysis

All data are presented as the mean ± SEM from three independent experiments. Differences were regarded as significant at *p* < 0.05 (^*^ *p* < 0.05, ^**^ *p* < 0.005). The significance of intergroup differences was evaluated through one‐way or two‐way ANOVA followed by Tukey's multiple comparison test and Student's *t*‐test for two‐component comparisons, after confirming a normal distribution.

### Code Availability

All codes generated in this study are available from the lead contact upon reasonable request.

## Conflict of Interest

The authors declare no conflict of interest.

## Author Contributions

B.C. and J.K. contributed equally to this work. B.C. and J.K. conceived and designed the project. S.K., S.A., Y.H., Y.K., and D.K., performed the experiments. J.K. supervised the study and obtained the financial supports and revised the manuscript. All the authors have read and approved the final manuscript.

## Supporting information



Supporting Information

## Data Availability

The data that support the findings of this study are available from the corresponding author upon reasonable request.
