# Supplementary material for: Epigenetic Dynamics in Reprogramming to Dopaminergic Neurons for Parkinson's Disease
Source: Adv Sci (Weinh). 2024 Sep 16;11(41):2403105. doi: 10.1002/advs.202403105 (PMC11538697; doi:10.1002/advs.202403105)
Supplement: Supplementary file 1 — Supporting Information [file ADVS-11-2403105-s001.docx]

**Epigenetic Dynamics in Reprogramming to Dopaminergic Neurons for Parkinson's Disease**

Byounggook Cho^1,#^, Junyeop Kim^1,#^, Sumin Kim^1^, Saemin An^1^, Yerim Hwang^1^, Yunkyung Kim^1^, Daeyeol Kwon^1^, Jongpil Kim^1*^

**Supplementary Information.**


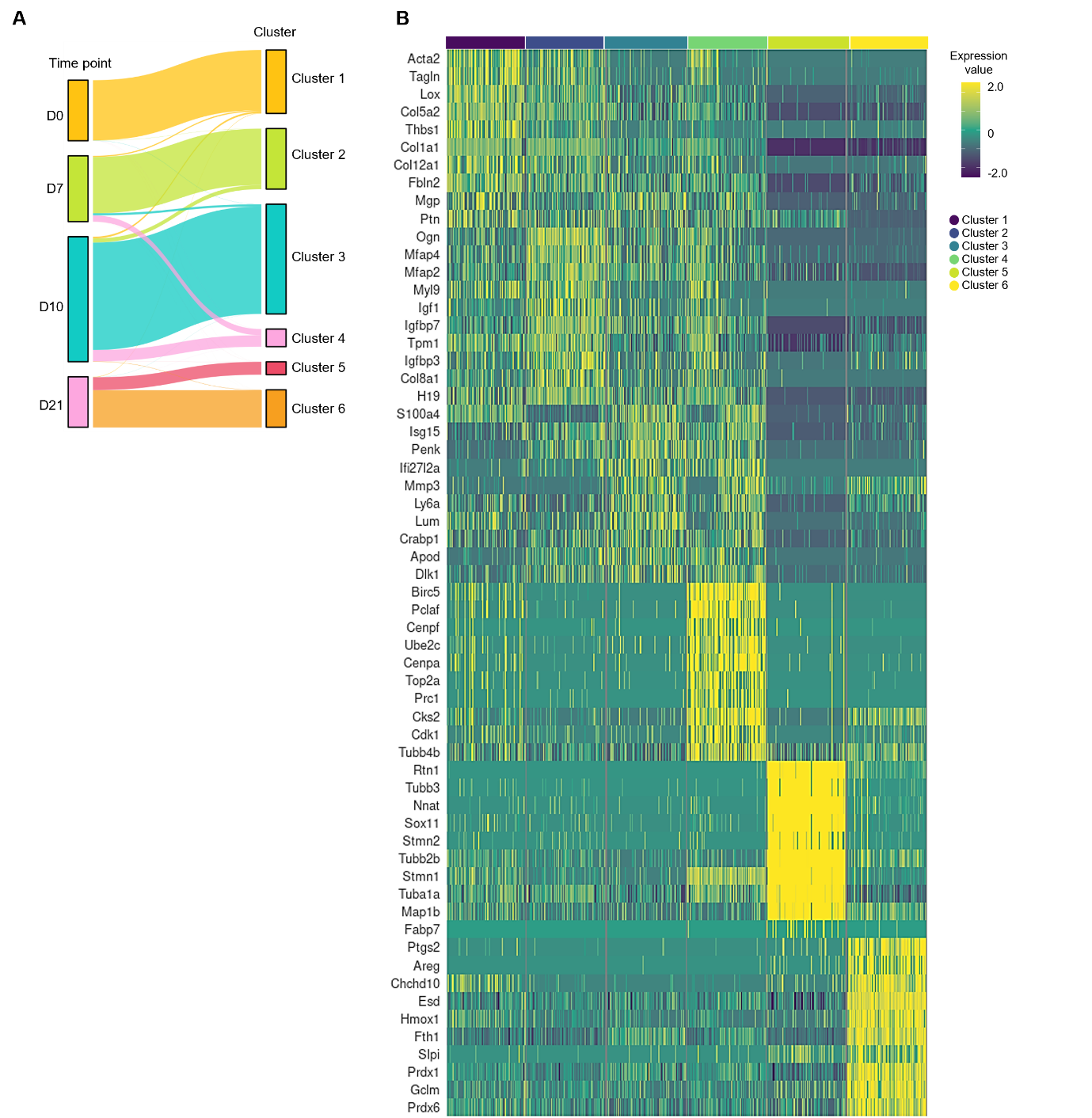


**Figure S1.**

(A) The Sankey plots of iDA reprogramming clustering results (Cluster 1 to Cluster 6) for the combined time-point dataset. (B) Hierachical clustering heatmap of top 10 highly expressed genes for each cluster.


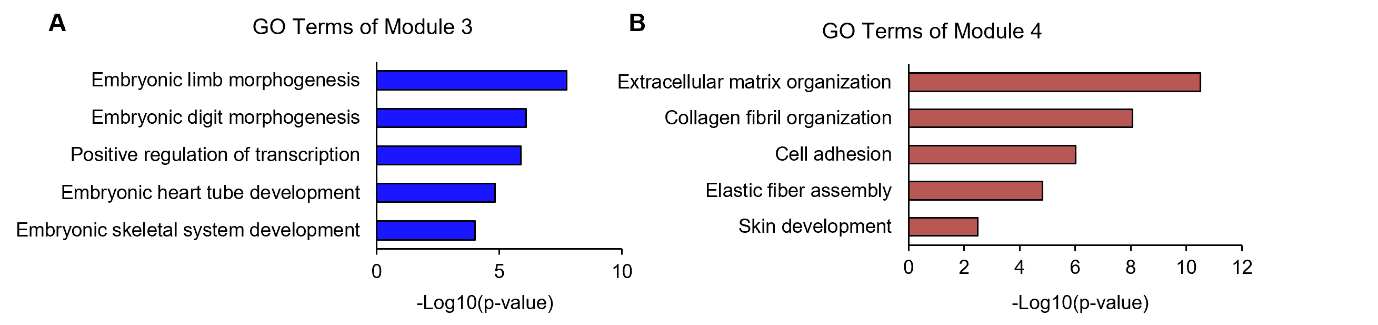


**Figure S2.**

(A) A bar graph displaying GO terms of Module 3. (B) A bar graph displaying GO terms of Module 4.


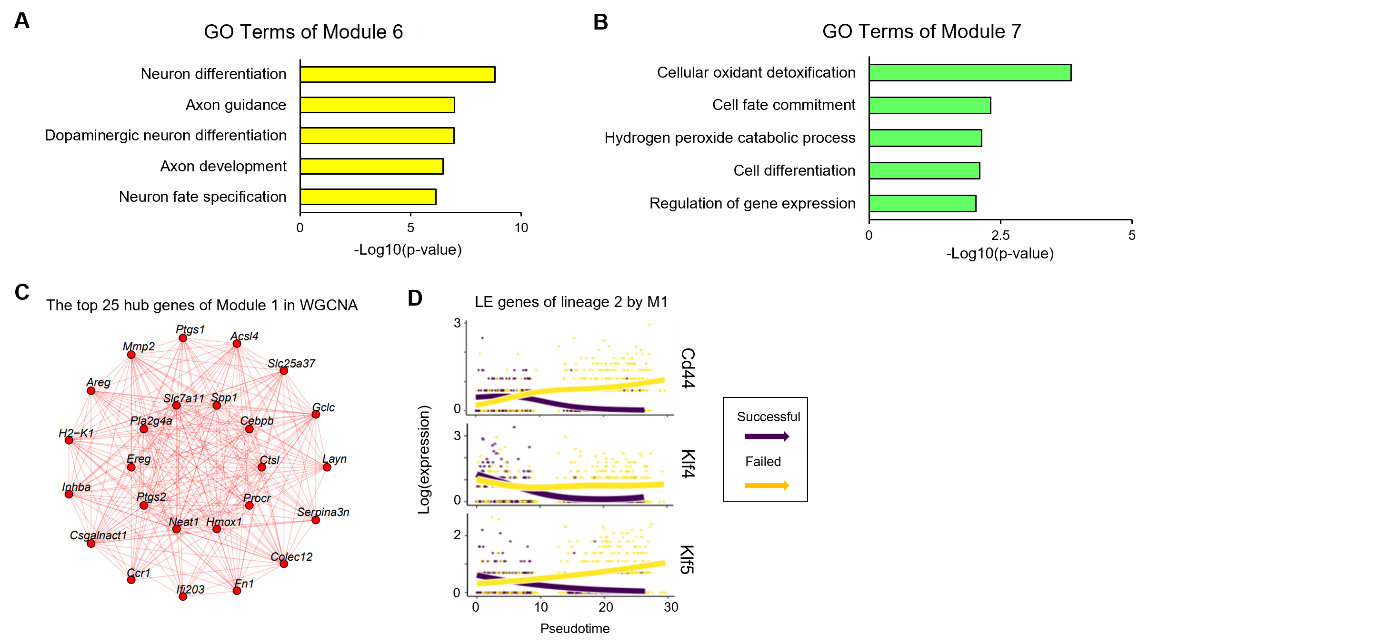


**Figure S3.**

(A) A bar graph displaying GO terms of Module 6. (B) A bar graph displaying GO terms of Module 7. (C) Network plot showing co-regulatory top 25 hub genes in module 1. (D) Visualization of smoothed expression patterns of three genes of module 1 in lineage 2 (Cluster 4 to 6).


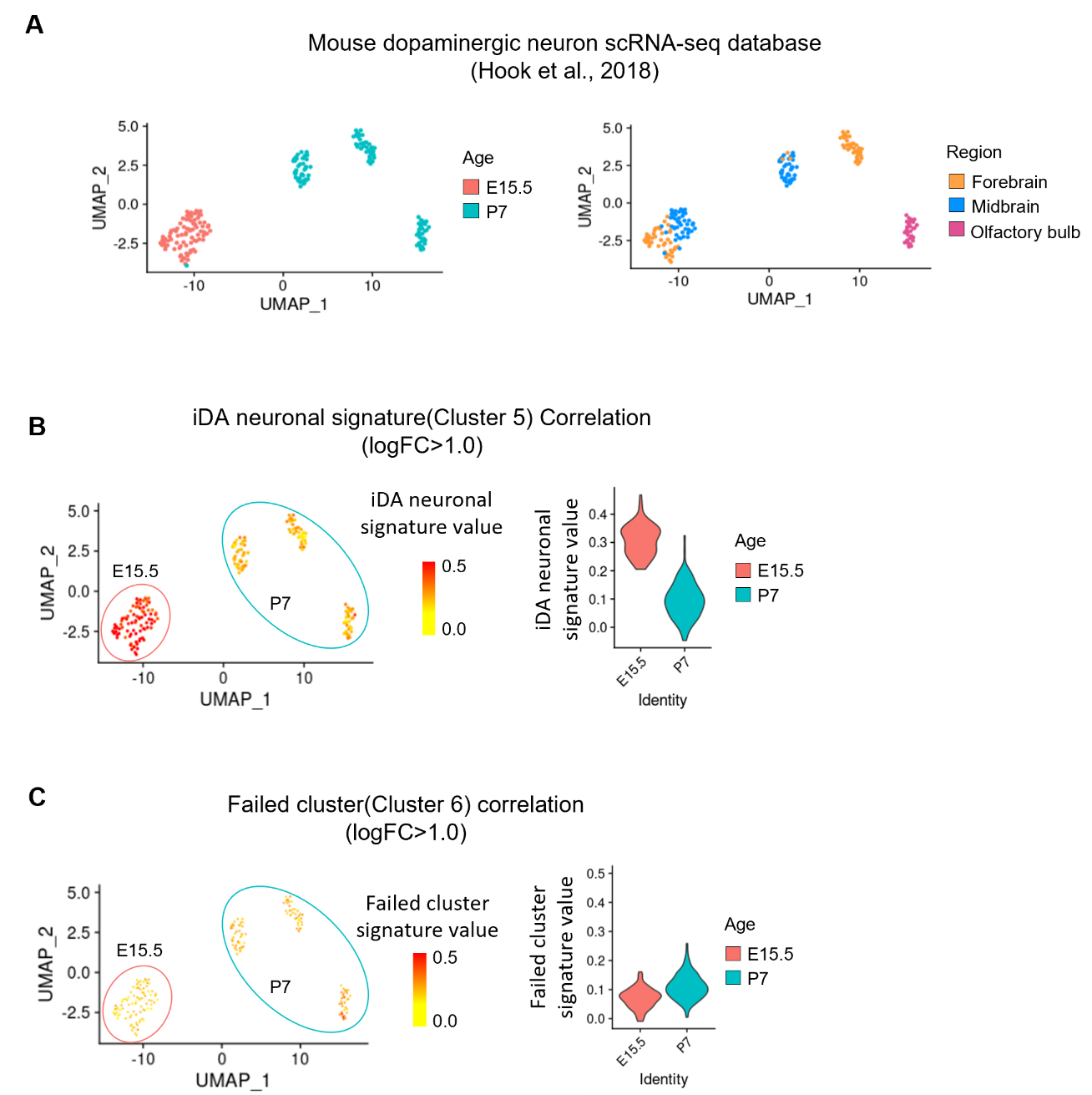


**Figure S4.**

(A) UMAP plot showing mouse dopaminergic neuron clusters across age (E15.5 and P7) and brain regions (forebrain, midbrain and olfactory bulb). (B) Correlation analysis of iDA cluster (Cluster 6) with public database of mouse dopaminergic neurons (E15.5 and P7) on UMAP projection and Violin plot. (C) UMAP and violin plot showing correlation score between failed reprogramming cluster (Cluster 5) and public sc-RNAseq database of mouse dopaminergic neurons (E15.5 and P7).


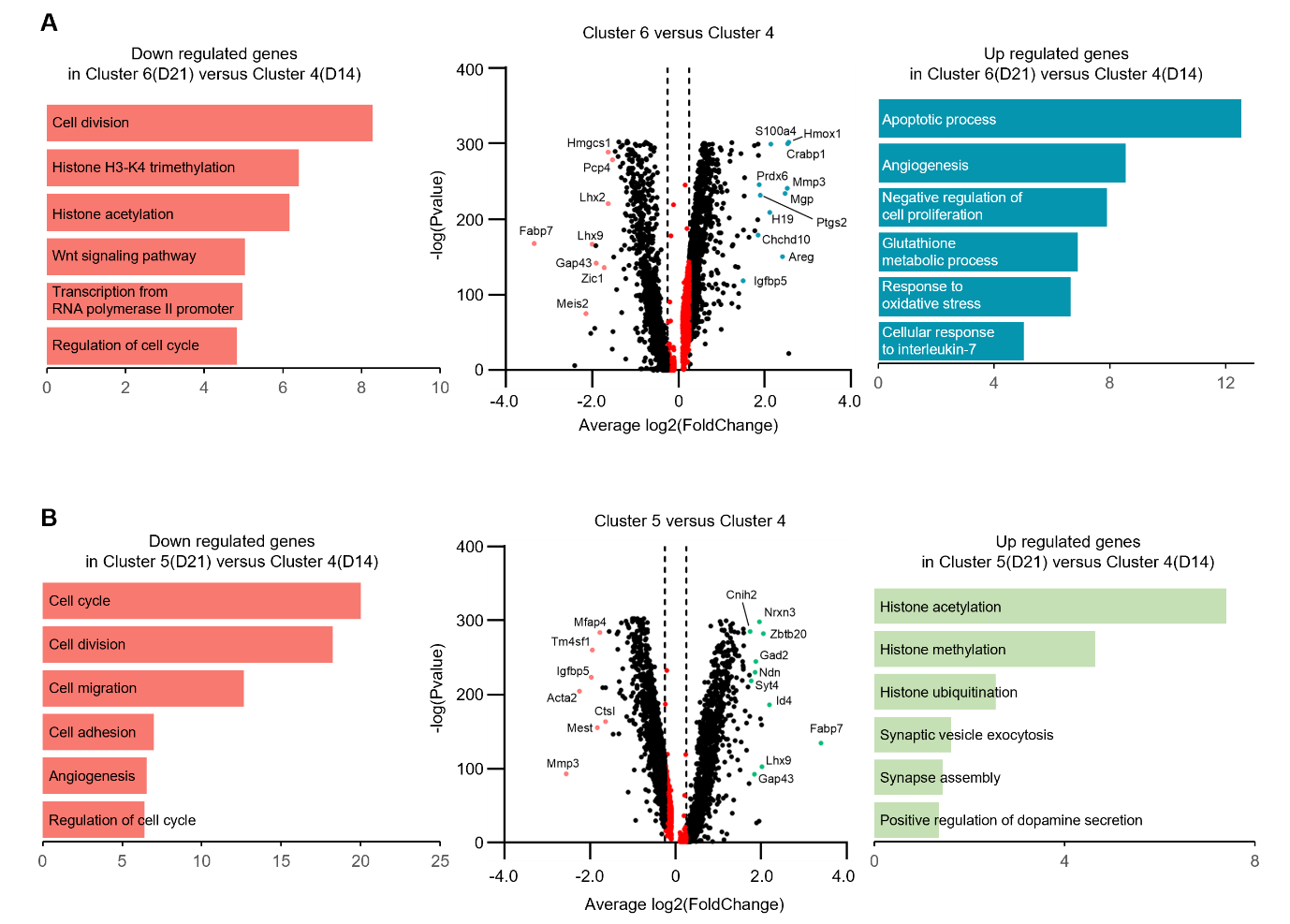


**Figure S5.**

(A) Volcano plot showing differentially expressed genes in Cluster 6 compared with Cluster 4 (Wilcoxon rank sum test; two-sided, log(fc) threshold > 0.1 and log(fc) threshold < -0.1). (B) Volcano plot showing differentially expressed genes in Cluster 5 compared with Cluster 4 (Wilcoxon rank sum test; two-sided, log(fc) threshold > 0.1 and log(fc) threshold < -0.1).


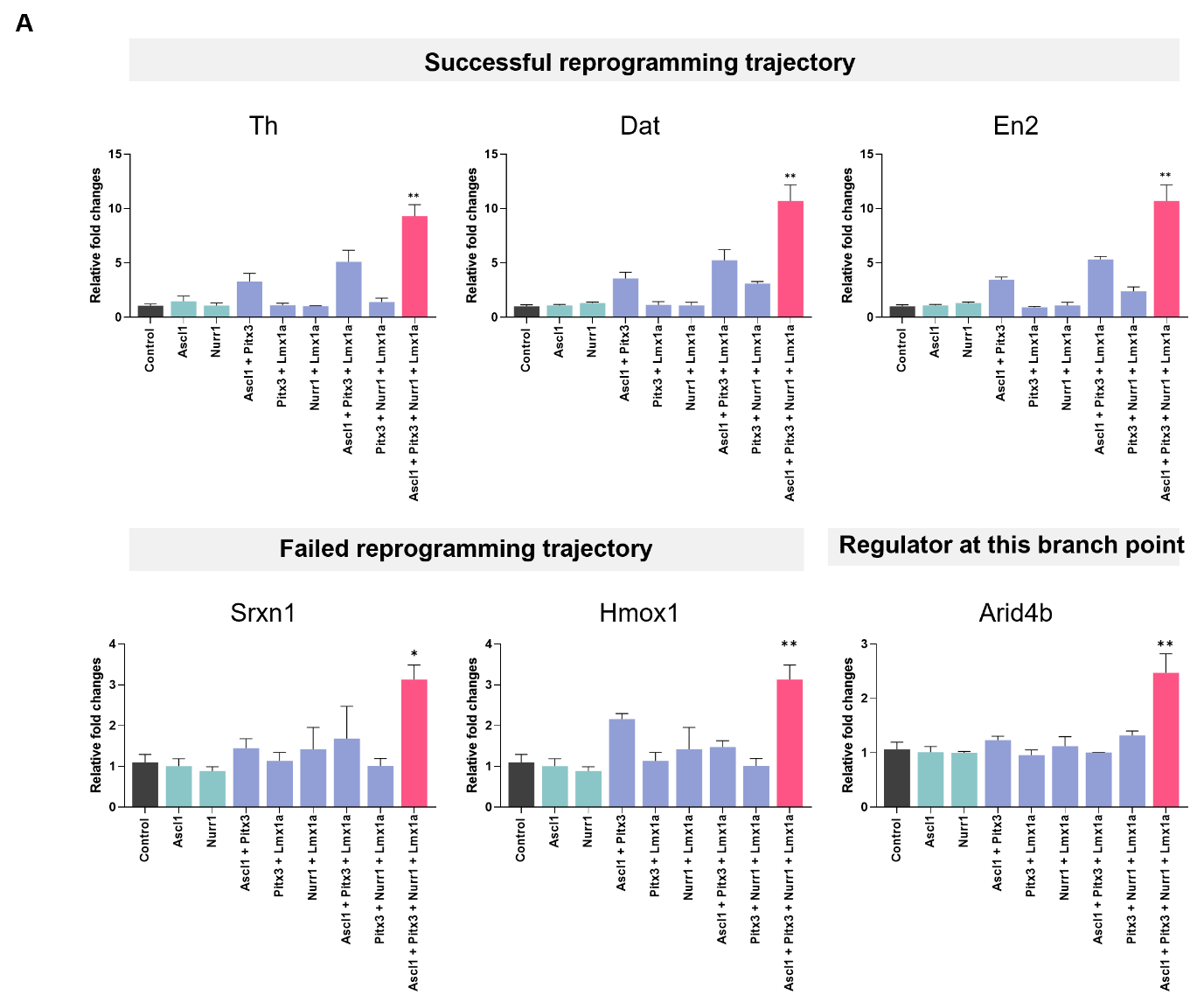


**Figure S6.**

(A) qRT-PCR analysis of successful iDA reprogramming markers (Th, Dat, En2 and Arid4b and failed reprogramming markers (Srxn1 and Hmox1) in mouse fibroblasts overexpressing various combinations of APNL. Expression levels are normalized to Gapdh. Data represent mean ± SEM; two-tailed Student's t test, *p < 0.05 and **p < 0.005 (n = 3, independent samples per group).


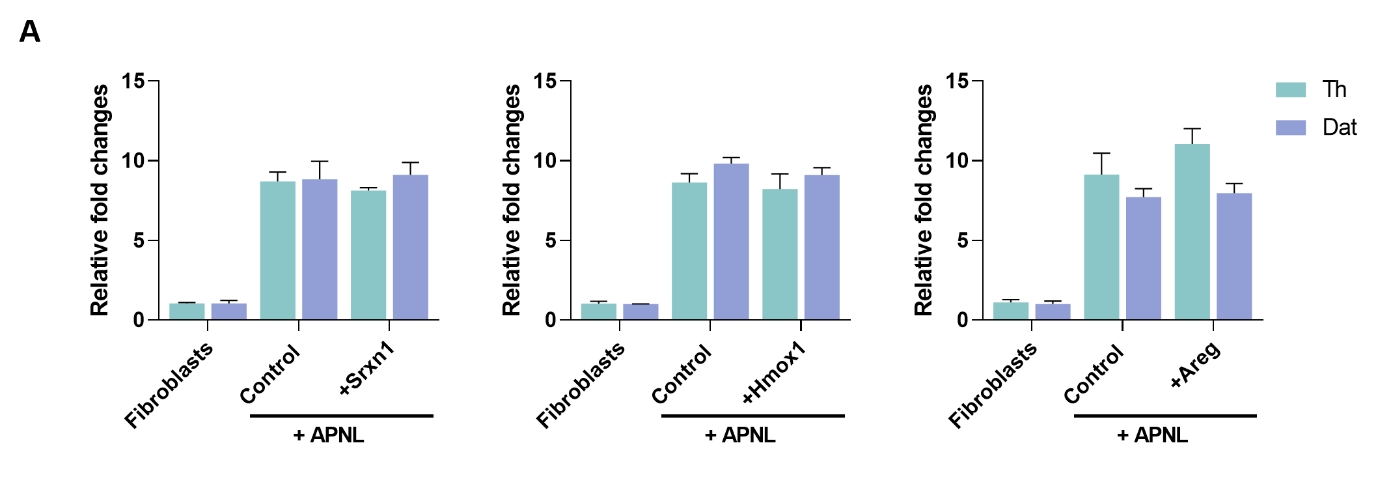


**Figure S7.**

(A) qRT-PCR analysis of DA neuronal markers (Th and Dat) in mouse fibroblasts overexpressing APNL along with Srxn1, Hmox1, and Areg. Expression levels are normalized to Gapdh. Data represent mean ± SEM; two-tailed Student's t test, *p < 0.05 and **p < 0.005 (n = 3, independent samples per group).


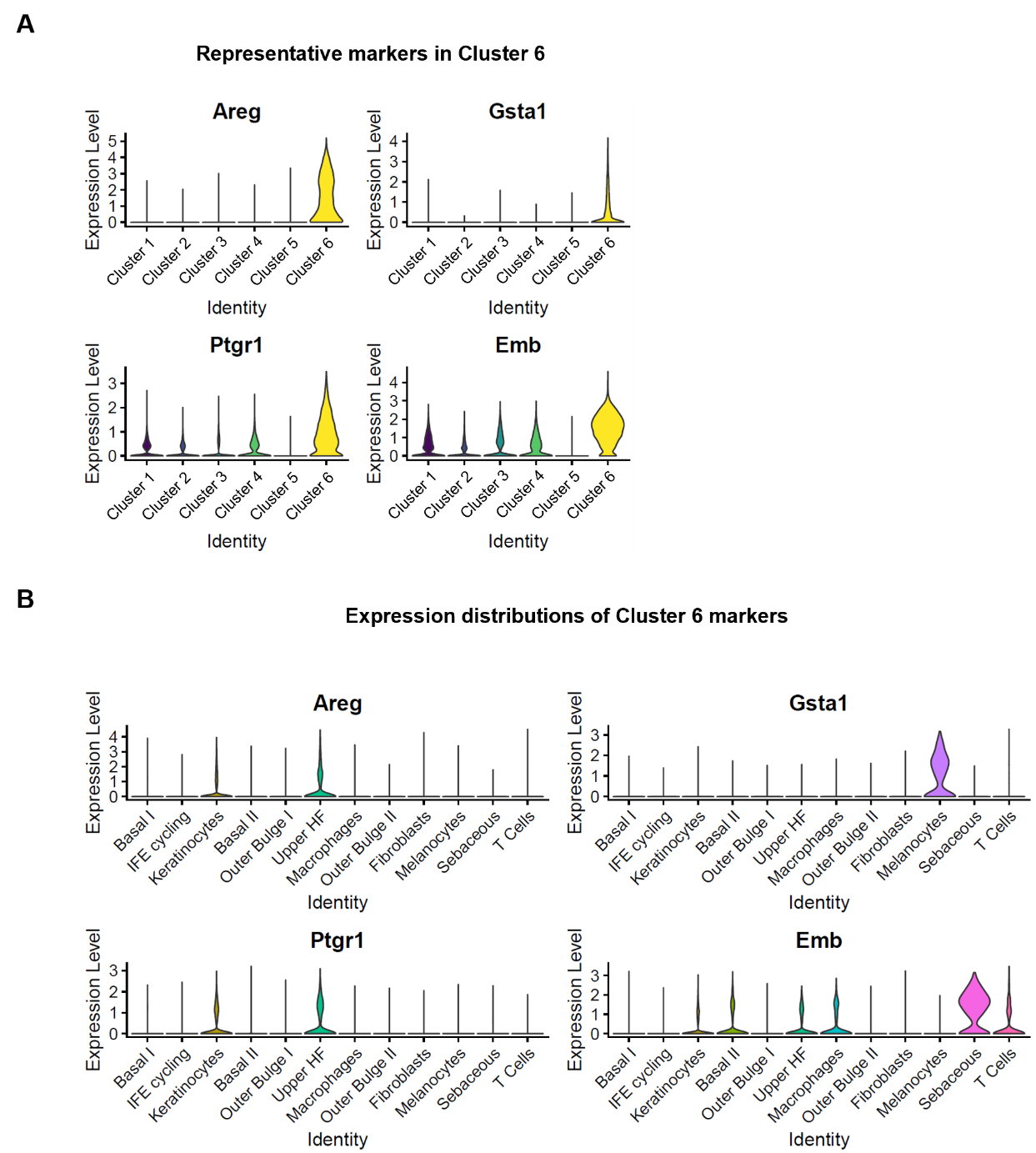


**Figure S8.**

(A) Violin plot showing highly expressed genes in Cluster 6. (B) Violin plot showing the expression levels of Cluster 6 markers in public sc-RNAseq data of mouse dorsal skin^40^.

**
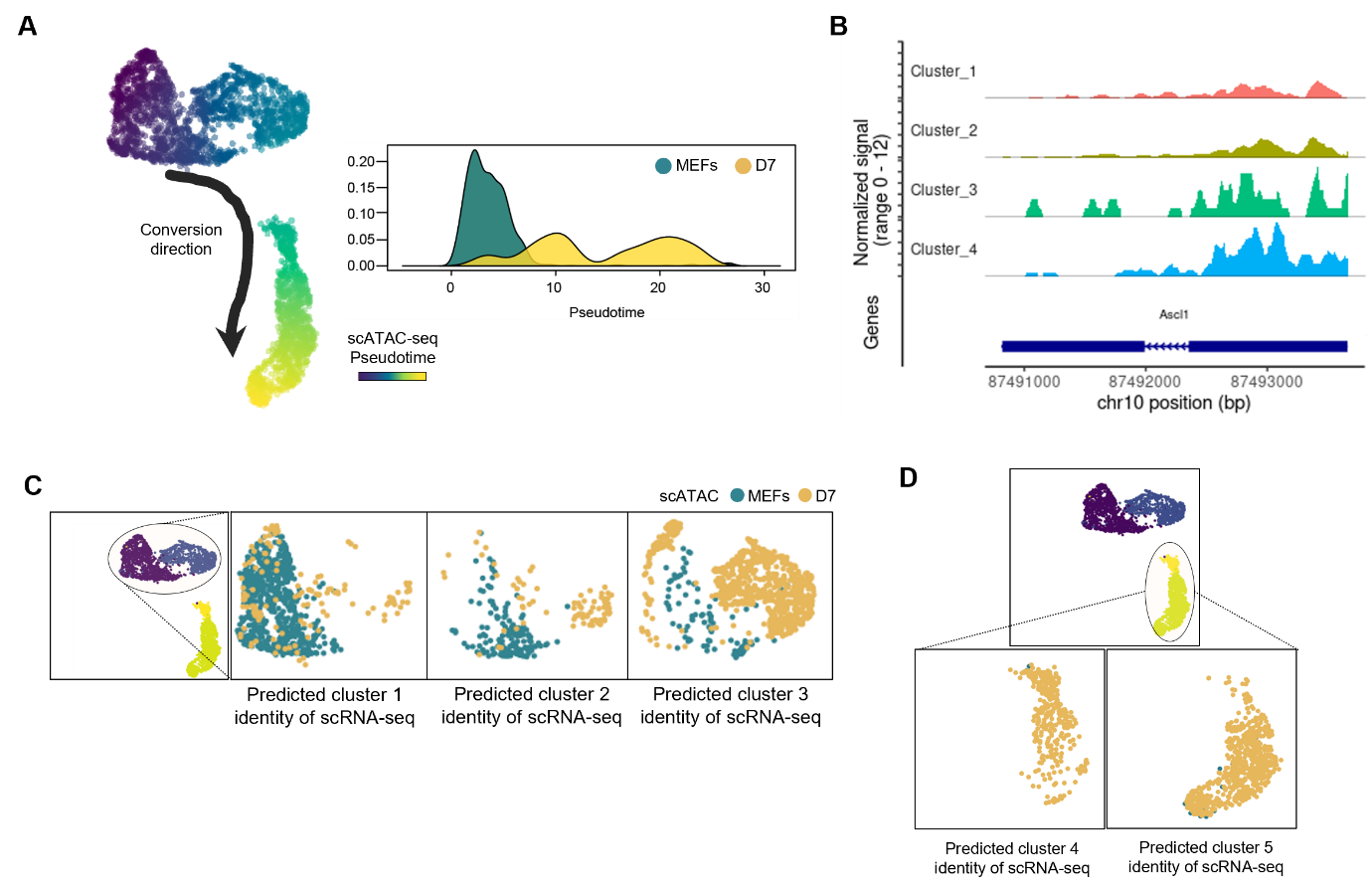
**

**Figure S9.**

(A) Continuous reprogramming trajectory of scATAC-seq by slingshot based UMAP plot (left). Density plot of reprogramming path of scATAC-seq across MEFs and D7 (right). (B) Coverage plot showing normalized open chromatin signal in each cluster on Ascl1 gene track. (C) UMAP plot showing predicted annotation results in Cluster 1 and 2 of scATAC-seq via label transfer from scRNA-seq data. (D) UMAP plot showing predicted annotation results in Cluster 3 and 4 of scATAC-seq via label transfer from scRNA-seq data.


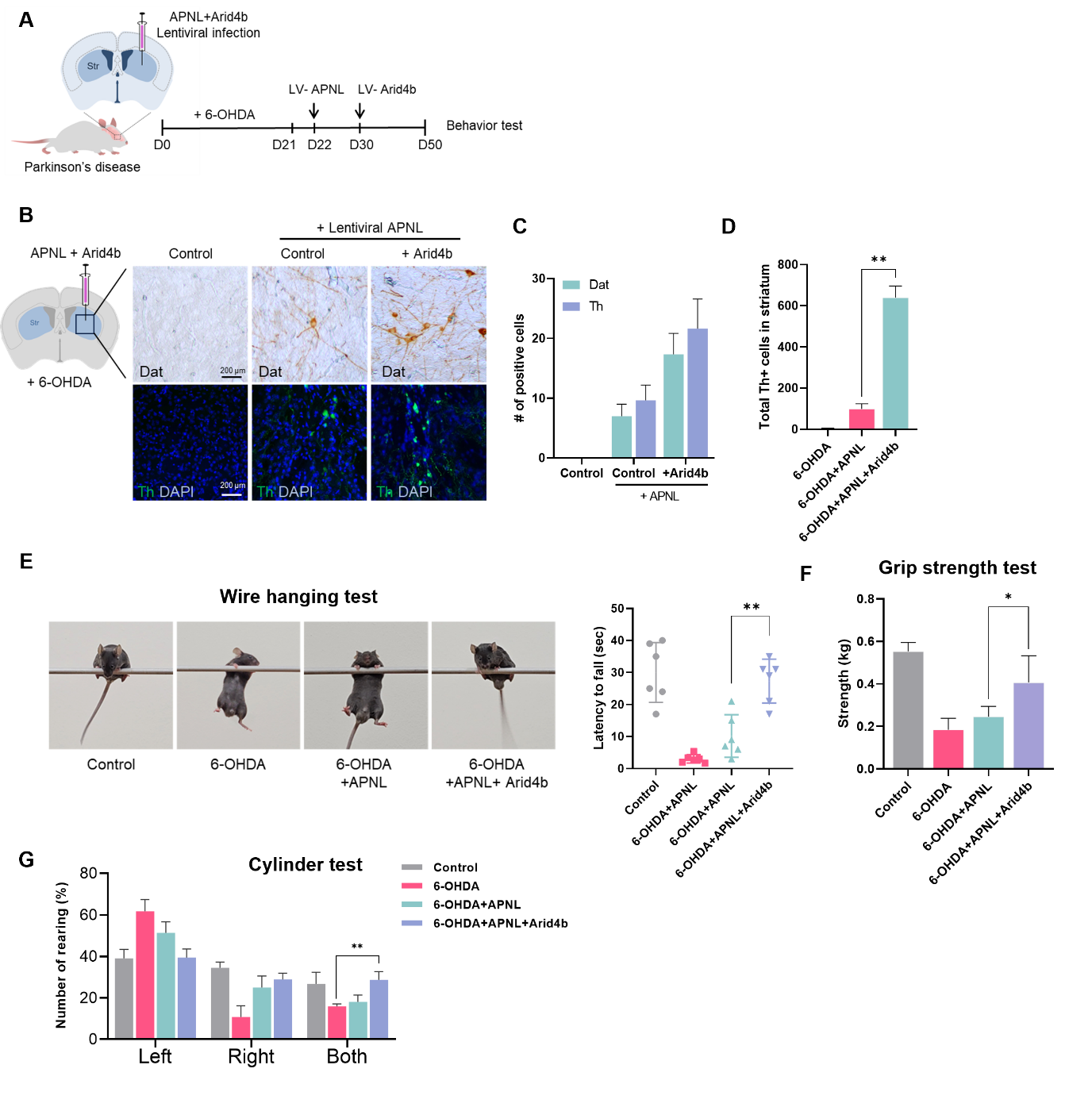


**Figure S10.**

(A) Schematic diagram depicts the procedures for direct reprogramming into induced dopaminergic neurons using Arid4b overexpression in the 6-OHDA-PD model. (B) Representative image of the DAB-Dat staining and immunostaining of Th in the stratum treated with Control (6-OHDA), Control (6-OHDA) + APNL, and Control (6-OHDA)+APNL+ Arid4b. Scale bar = 200 μm. (C) The bar chart shows the quantification data of number of positive cells. Data represent mean ± SEM; two-tailed Student's t test, *p < 0.05 and **p < 0.005 (n = 3, independent samples per group). (D) Number of Th positive cells in the entire striatum region of mouse brains. (E) 6-OHDA PD mouse behaviors after overexpression of APNL with Arid4b in the wire-hanging test. The latency to fall in the wire-hanging test (Right). (F) Values were measured using the forelimb grip strength test in mice treated with 6-OHDA, 6-OHDA/APNL, 6-OHDA/Arid4b, and 6-OHDA/ANPL/Arid4b. (G) Limb use asymmetry was assessed using the cylinder test for the right, left, and both forepaws. Data represent mean ± SEM; Two-way ANOVA, *p < 0.0332 and **p < 0.0021 (n = 3, independent samples per group).
